# Supplementary material for: Stepwise ATP translocation into the endoplasmic reticulum by human SLC35B1
Source: Nature. 2025 May 21;643(8072):855–64. doi: 10.1038/s41586-025-09069-w (PMC12267056; doi:10.1038/s41586-025-09069-w)
Supplement: Supplementary file 1 — Supplementary Figs. 1–8, Table 1 and references. [file 41586_2025_9069_MOESM1_ESM.pdf]

nature portfolio

https://doi.org/10.1038/s41586-025-09069-w

Supplementary information

Stepwise ATP translocation into the endoplasmic reticulum by human SLC35B1

In the format provided by the authors and unedited

	T_M (°C) SLC35B1 + buffer	T_M (°C) SLC35B1 + ATP	ΔT_M (°C)
**WT**	33.8 ± 0.4	39.0 ± 0.6	**5.2**
**Y25A**	32.9 ± 1.1	35.9 ± 1.2	**3.0**
**E33A**	30.7 ± 0.4	38.3 ± 0.5	**7.6**
**R37A**	27.0 ± 1.1	28.5 ± 1.0	**1.5**
**Y94A**	34.1 ± 0.5	38.4 ± 0.6	**4.3**
**Y110A**	32.7 ± 1.4	36.5 ± 1.0	**3.8**
**Q113E**	33.3 ± 0.8	36.1 ± 0.9	**2.8**
**Q113F**	37.2 ± 1.2	43.8 ± 0.7	**6.6**
**K117A**	35.0 ± 1.2	36.8 ± 1.4	**1.8**
**K120A**	34.0 ± 0.6	35.4 ± 0.8	**1.4**
**Q190A**	35.3 ± 1.2	41.5 ± 0.7	**6.2**
**R194A**	35.3 ± 0.5	38.2 ± 0.7	**2.9**
**Q254A**	32.1 ± 0.4	35.6 ± 0.6	**3.5**
**I257E**	35.9 ± 0.5	37.6 ± 0.5	**1.7**
**I257F**	31.2 ± 0.4	35.2 ± 0.6	**4.0**
**V261T**	33.9 ± 0.4	35.9 ± 0.4	**2.0**
**C269A**	36.3 ± 0.4	39.3 ± 0.4	**3.0**
**C269S**	30.6 ± 0.4	34.8 ± 0.6	**4.2**
**T273A**	33.2 ± 0.3	35.8 ± 0.4	**2.6**
**R276A**	38.3 ± 0.7	38.6 ± 0.9	**0.3**
**K277A**	34.7 ± 0.4	37.0 ± 1.0	**2.3**

Supplementary Table 1. Apparent T_M melting temperatures of SLC35B1 and variants with and without ATP and the difference (ΔT_M) as determined by GFP-TS using a range of 8 different temperatures that were fitted to a sigmoidal dose-response equation as described in Methods. Each apparent T_M is the mean ± s.d. of the fit from n = 3 independent titrations.

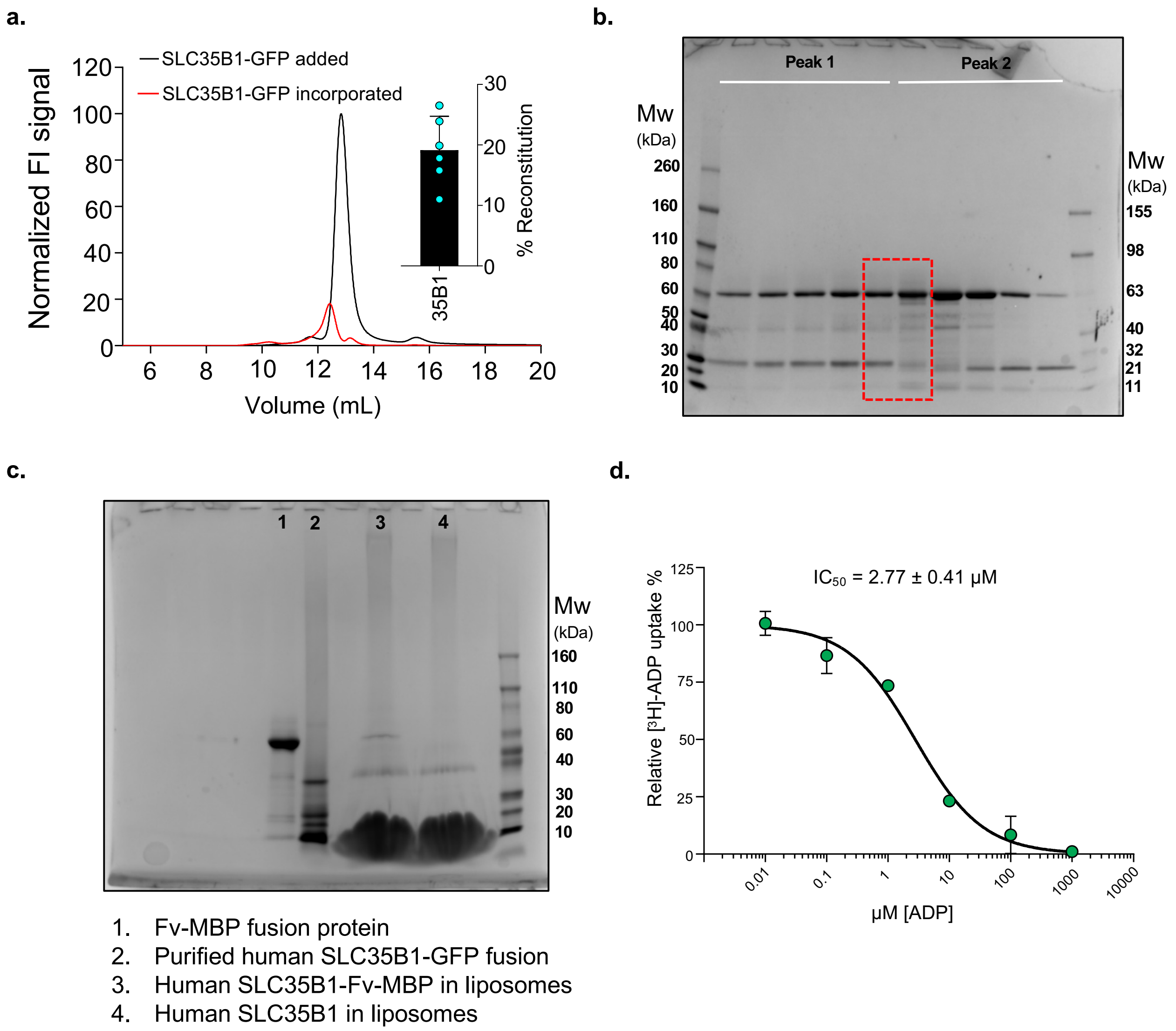

Supplementary Fig. 1. Reconstitution efficiency of SLC35B1 into liposomes a, Representative FSEC traces of detergent-solubilized proteoliposomes containing SLC35B1. The fluorescence signal was normalized to the total amount of purified SLC35B1-GFP used in the reconstitutions. *insert*: bar graph showing the reconstitution efficiency calculated from the total fluorescence SLC35B1-GFP peak area. Error bars are the mean ± s.d. of n = 6 independent reconstitutions. **b,** Coomassie-stained SDS-PAGE of the SEC purified SLC35B1 mixed with excess Fv-MBP fusion protein. **c**, Coomassie-stained SDS-PAGE of SLC35B1 showing that the reconstitution efficiency with and without the Fv-MBP fusion protein is similar. **d,** IC_{50} curves for external ADP competition of [^{3}H]-ADP/ATP normalized transport of SLC35B1-Fv-MBP fusion protein. Error bars are the mean ± s.d. of n= 3 independent experiments from two separate reconstitutions.

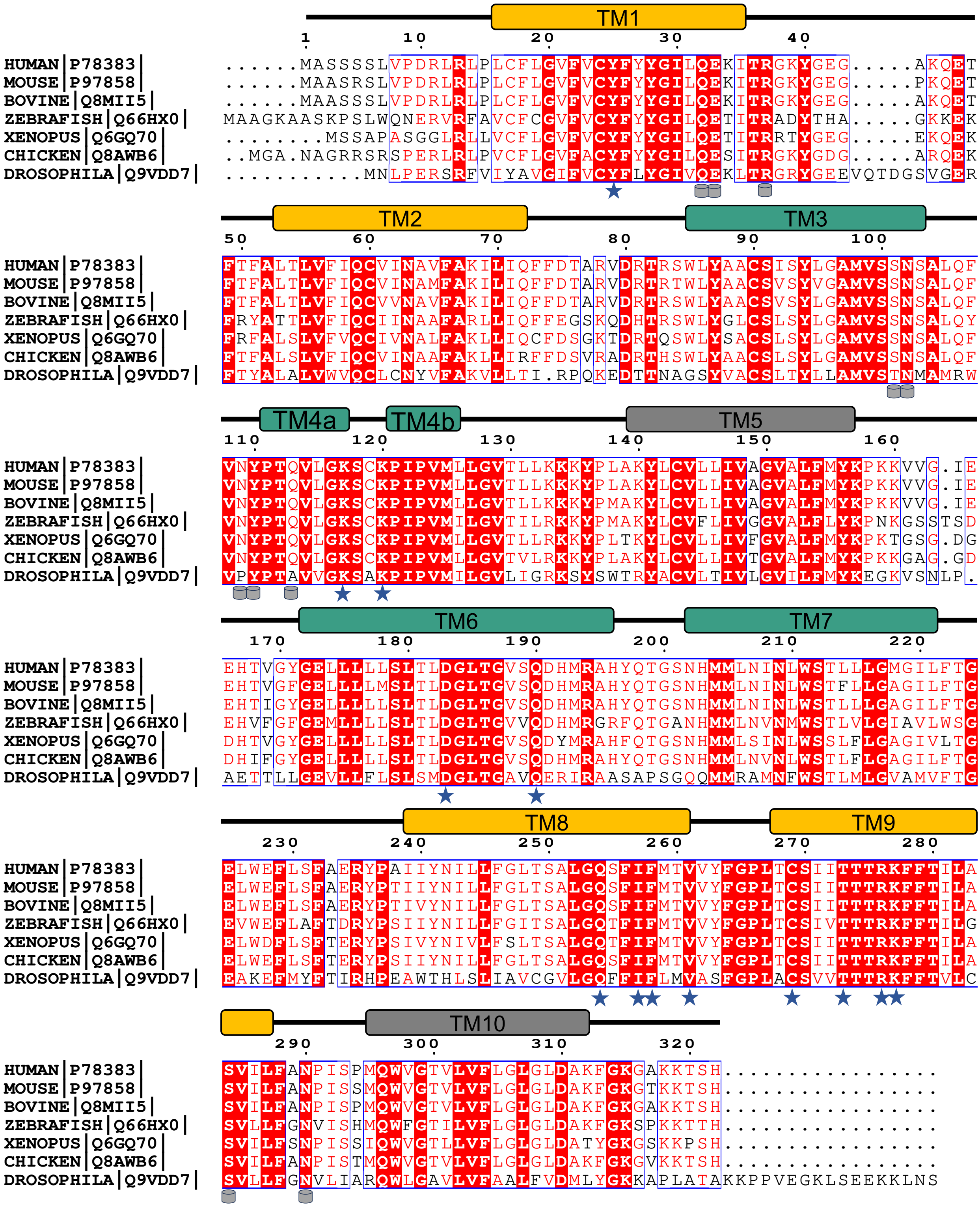

Organism	Mouse	Bovine	Zebrafish	Xenopus	Chicken	Drosphilla
% identity with human	95.6	97.6	72.7	80.9	87.9	50.2

Supplementary Fig. 2. Multiple amino acid sequence alignment of SLC35B1. *above*: Amino acid sequence alignment of divergent SLC35B1 members with the corresponding position of TM helices labelled and coloured as in the SLC35B1 structure shown in Fig. 1b. Uniprot identifiers of SLC35B1 are shown. Conserved residues coordinating the nucleotide are marked with a blue star and conserved ER luminal cavity-closing contacts as grey cylinders. *below*; Table showing the amino acid sequence identity (%) between SLC35B1 homologues shown in the alignment, which was generated using Clustal Omega[1] and rendered using Espript3.0.

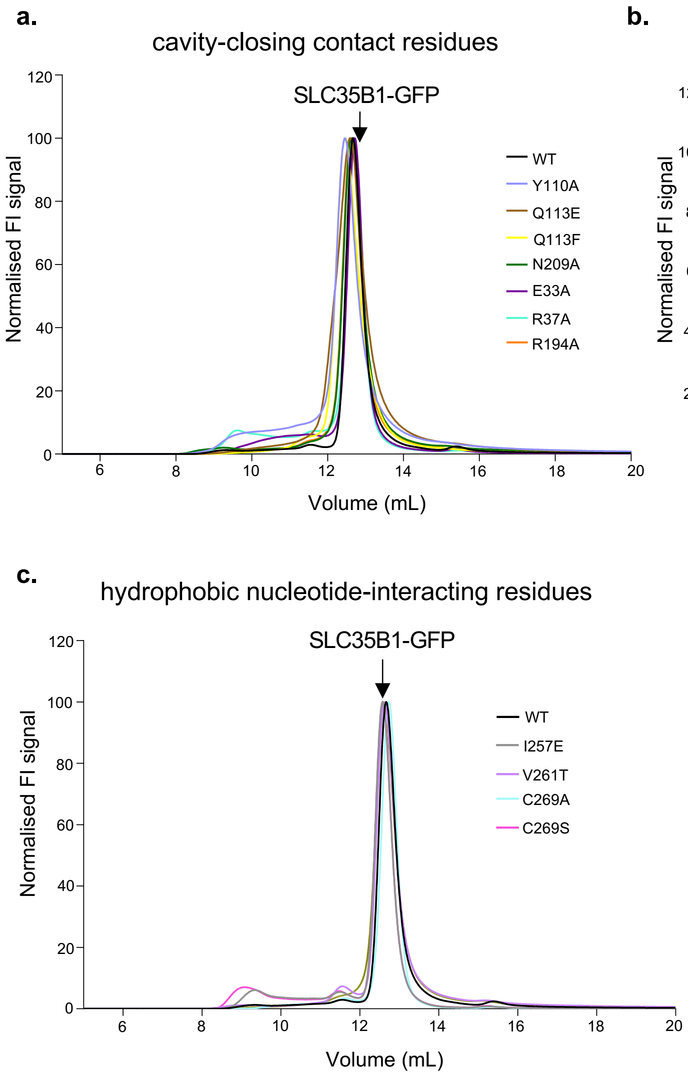

Supplementary Fig. 3. Evaluation of purified SLC35B1 variants. a. FSEC traces of purified SLC35B1 WT and variants of residues forming cavity-closing contacts that were analysed on an Enrich SEC 650 10 x 300 column (Biorad). All FSEC traces were normalized to their own maximal peak intensity. **b**. as in a, for polar nucleotide-interacting residues. **c**. as in a., for hydrophobic nucleotide-interacting residues.

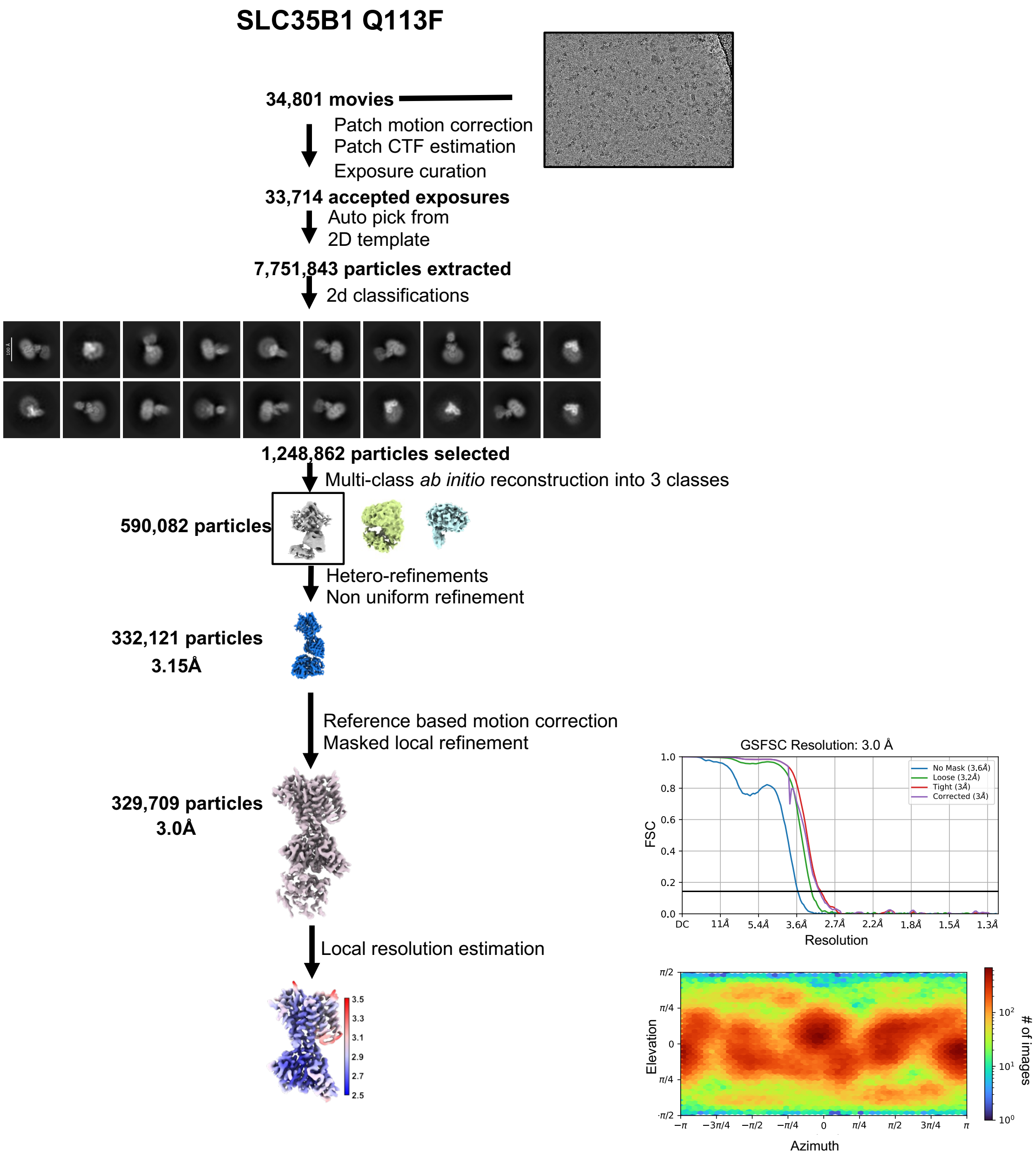

Supplementary Fig. 4. Cryo-EM processing workflow of SLC35B1 Q113F with AMP-PNP. Data was processed using CryoSPARC[2]. Movie frames were aligned using the "Patch motion correction" and contrast transfer function was estimated using the "patch CTF" algorithms. Dataset was pruned using multiple rounds of 2D classifications, initial maps were generated using multiclass *ab initio* reconstruction and cleaned using heterogenous refinement. Final Q113F cryo EM maps with AMP-PNP were reconstructed from 329,709 particles after local refinement with MBP masking, with an overall resolution of 3.0 Å resolution according to the FSC at 0.143. Volumes were rendered using ChimeraX[3].

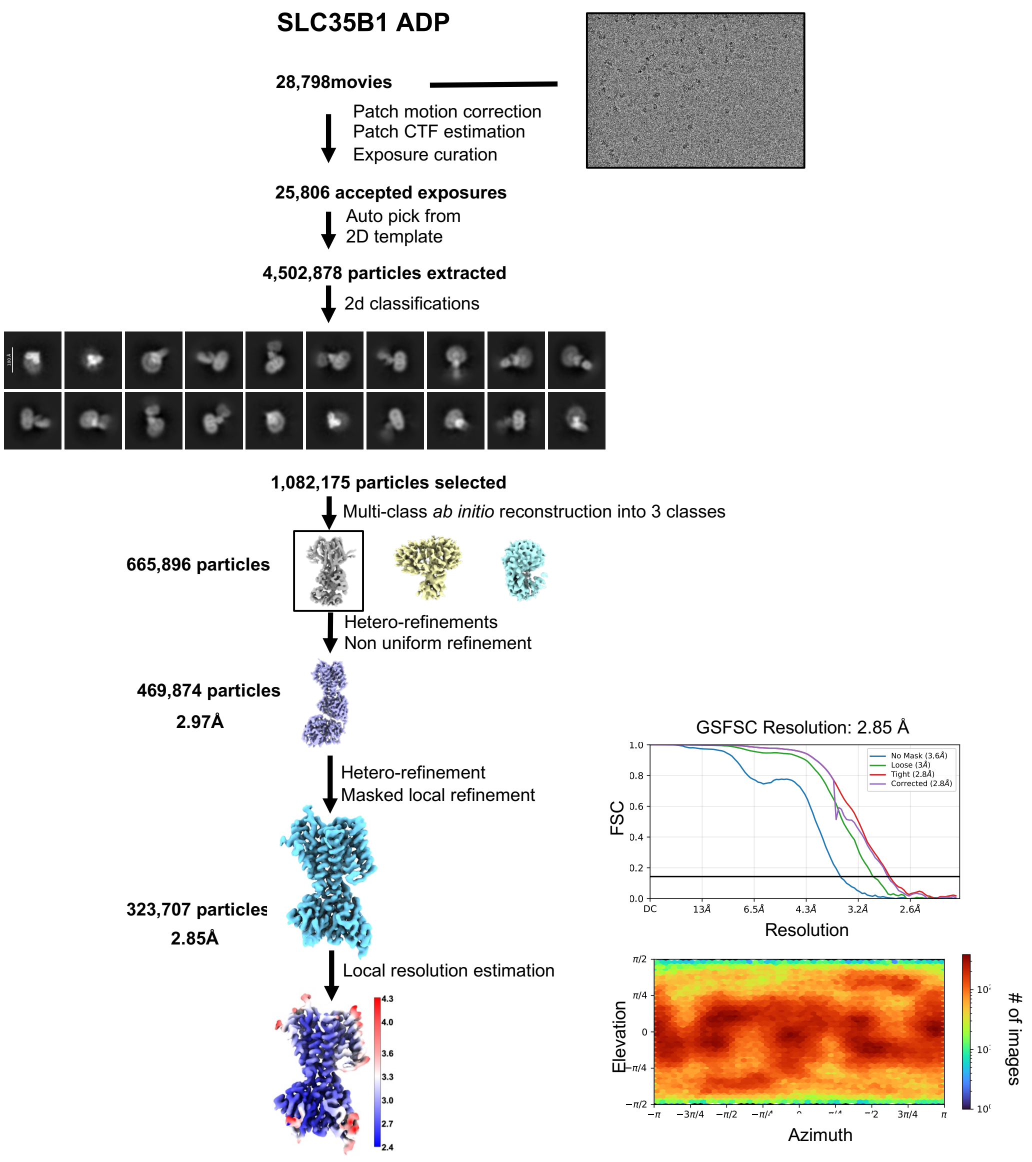

Supplementary Fig. 5. Cryo-EM processing workflow of SLC35B1 WT with ADP. The dataset was processed using CryoSPARC[2]. Movie frames were aligned using the "Patch motion correction" and contrast transfer function was estimated using the "patch CTF" algorithms. Dataset was pruned using multiple rounds of 2D classifications, initial maps were generated using multiclass *ab initio* reconstruction and cleaned using heterogenous refinement. Final WT cryo EM maps with ADP were reconstructed from 323,707 particles after local refinement with MBP masking, with an overall resolution of 2.85 Å resolution according to the FSC at 0.143. Volumes were rendered using ChimeraX[3].

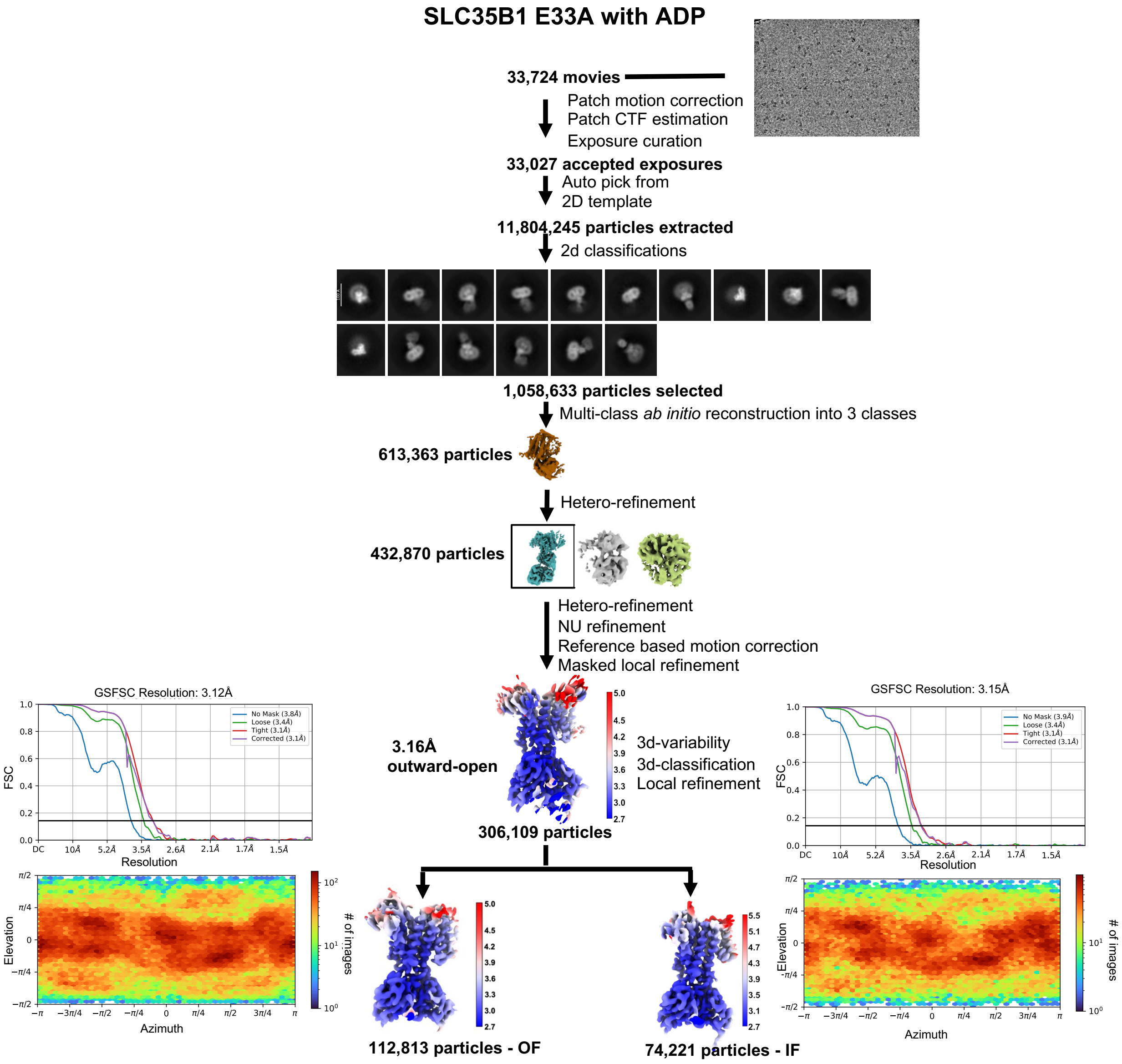

Supplementary Fig. 6. Cryo-EM processing workflow of SLC35B1 E33A with ADP. The dataset was processed using CryoSPARC[2]. Movie frames were aligned using the "Patch motion correction" and contrast transfer function was estimated using the "patch CTF" algorithms. Dataset was pruned using multiple rounds of 2D classifications, initial maps were generated using multiclass *ab initio* reconstruction and cleaned using heterogenous refinement. A 3.16 Å reconstruction in the outward facing state was obtained from 306,109 particles using NU and local refinement. After 3D variability analysis, a separate inward-facing state was obtained after 3D classification without alignment. Final luminal-facing (OF) and cytoplasmic-facing (IF) reconstructions had an overall estimated resolution of 3.12 Å and 3.15 Å based on FSC at 0.143, respectively. Volumes were rendered using ChimeraX[3].

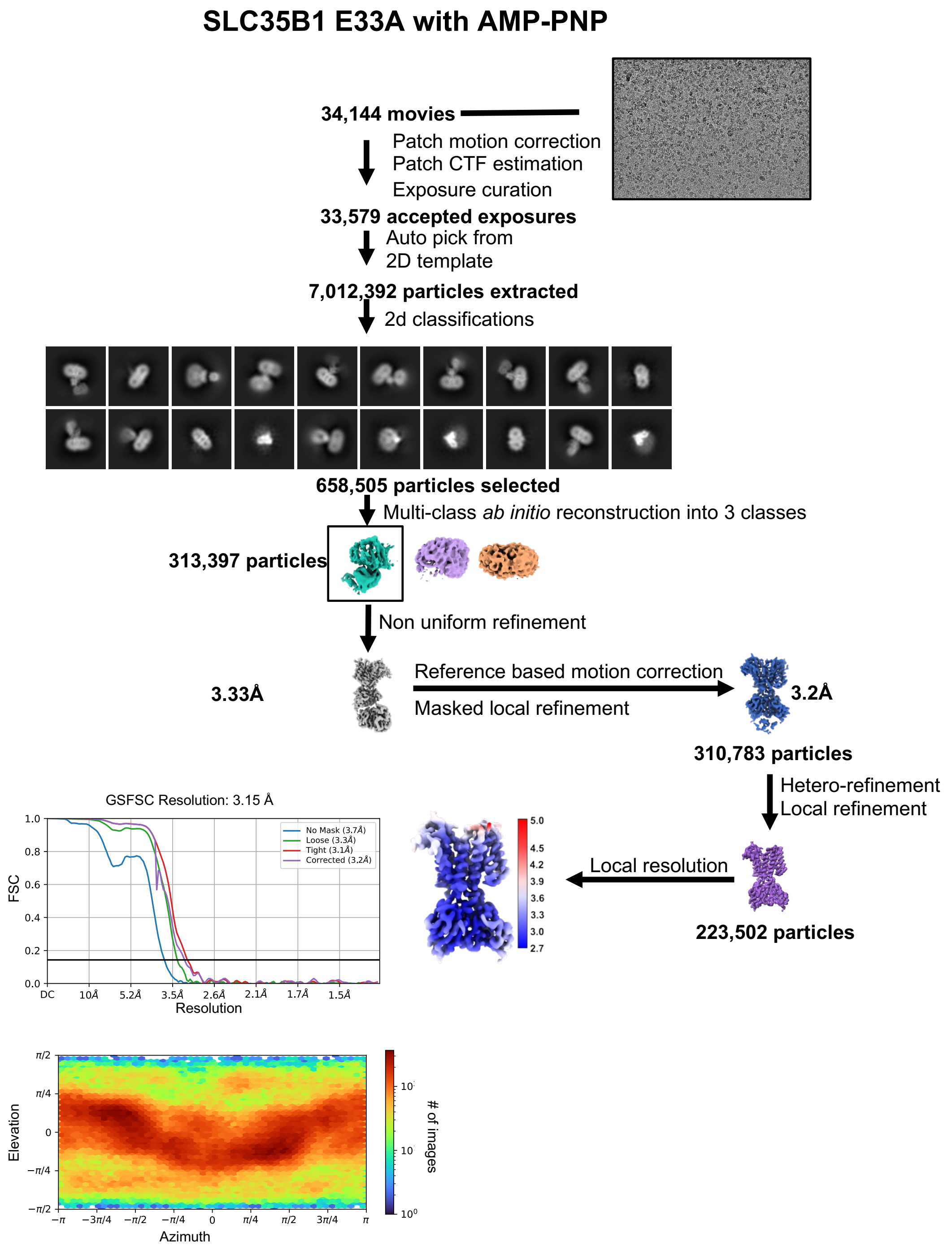

Supplementary Fig. 7. Cryo-EM processing workflow of SLC35B1 E33A variant in the presence of AMP-PNP. The dataset was processed CryoSPARC[2]. Movie frames were aligned using the "Patch motion correction" and contrast transfer function was estimated using the "patch CTF" algorithms. Dataset was pruned using multiple rounds of 2D classifications, initial maps were generated using multiclass *ab initio* reconstruction and cleaned using heterogenous refinement. Final E33A cryo EM maps with AMP-PNP were reconstructed from 223,502

particles after local refinement with MBP masking, with an overall resolution of 3.15 Å resolution according to the FSC at 0.143. Volumes were rendered using ChimeraX[3].

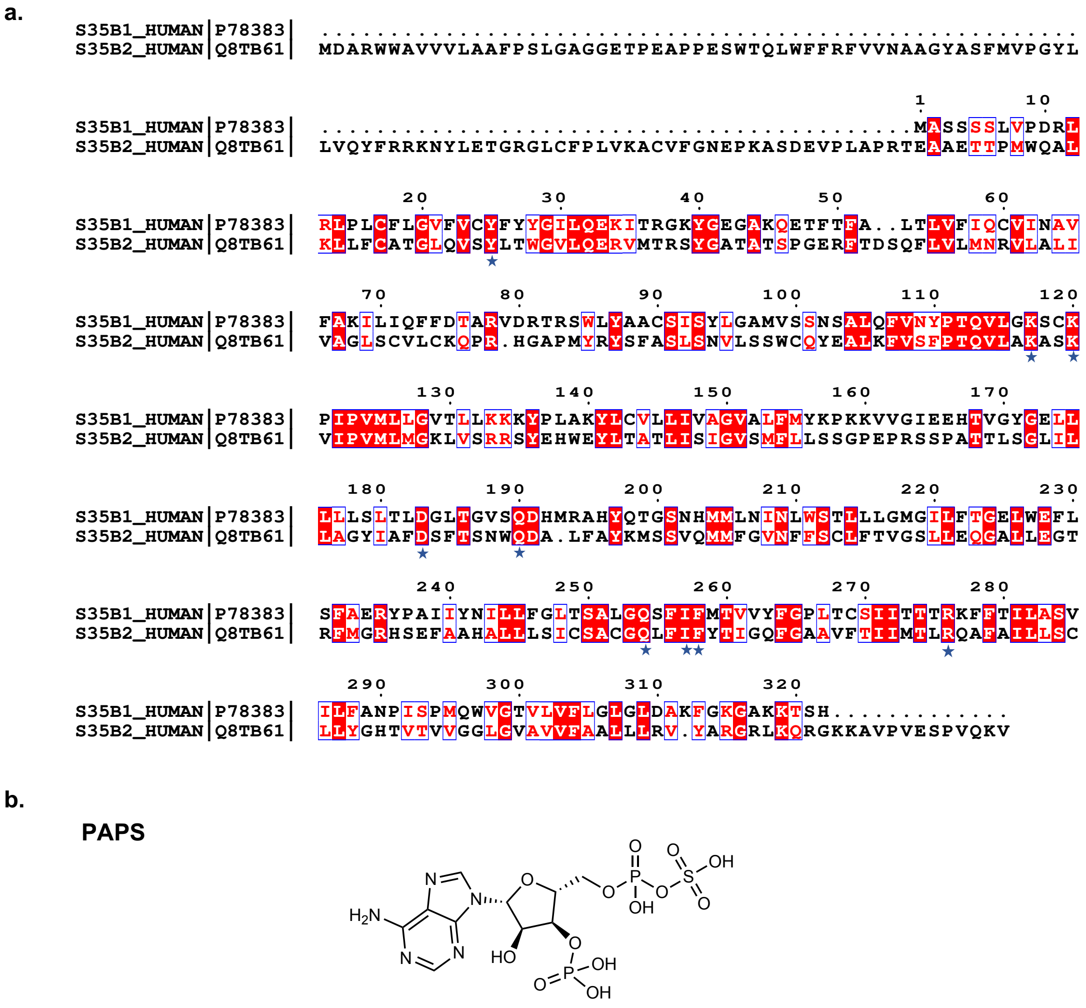

Supplementary Fig. 8. a, Sequence alignment of *human* SLC35B1 and SLC35B2. Uniprot identifiers for the respective protein sequences are shown. SLC35B2 transport PAPS, a universal sulphate donor synthesized from ATP. Conserved nucleotide-binding residues are marked with a blue star. **b,** Chemical structure of 3′-phosphoadenosine-5′-phosphosulfate (PAPS), which is transported by SLC35B2[4].

1 Madeira, F. *et al.* The EMBL-EBI Job Dispatcher sequence analysis tools framework in 2024. *Nucleic Acids Res* **52**, W521-W525 (2024). https://doi.org:10.1093/nar/gkae241

2 Punjani, A., Rubinstein, J. L., Fleet, D. J. & Brubaker, M. A. cryoSPARC: algorithms for rapid unsupervised cryo-EM structure determination. *Nat Methods* **14**, 290-296 (2017). https://doi.org:10.1038/nmeth.4169

3 Meng, E. C. *et al.* UCSF ChimeraX: Tools for structure building and analysis. *Protein science : a publication of the Protein Society* **32**, e4792 (2023). https://doi.org:10.1002/pro.4792

4 Kamiyama, S. *et al.* Molecular cloning and identification of 3'-phosphoadenosine 5'-phosphosulfate transporter. *J Biol Chem* **278**, 25958-25963 (2003). https://doi.org:10.1074/jbc.M302439200
