# Supplementary material for: Stepwise ATP translocation into the endoplasmic reticulum by human SLC35B1
Source: Nature. 2025 May 21;643(8072):855–64. doi: 10.1038/s41586-025-09069-w (PMC12267056; doi:10.1038/s41586-025-09069-w)
Supplement: Supplementary file 2 — Reporting Summary [file 41586_2025_9069_MOESM2_ESM.pdf]

nature portfolio

Corresponding author(s): David Drew

Last updated by author(s): Apr 7, 2025

Reporting Summary

Nature Portfolio wishes to improve the reproducibility of the work that we publish. This form provides structure for consistency and transparency in reporting. For further information on Nature Portfolio policies, see our Editorial Policies and the Editorial Policy Checklist.

Statistics

For all statistical analyses, confirm that the following items are present in the figure legend, table legend, main text, or Methods section.

n/a	Confirmed	
☐	☒	The exact sample size (*n*) for each experimental group/condition, given as a discrete number and unit of measurement
☐	☒	A statement on whether measurements were taken from distinct samples or whether the same sample was measured repeatedly
☒	☐	The statistical test(s) used AND whether they are one- or two-sided<br>*Only common tests should be described solely by name; describe more complex techniques in the Methods section.*
☒	☐	A description of all covariates tested
☒	☐	A description of any assumptions or corrections, such as tests of normality and adjustment for multiple comparisons
☐	☒	A full description of the statistical parameters including central tendency (e.g. means) or other basic estimates (e.g. regression coefficient) AND variation (e.g. standard deviation) or associated estimates of uncertainty (e.g. confidence intervals)
☒	☐	For null hypothesis testing, the test statistic (e.g. *F*, *t*, *r*) with confidence intervals, effect sizes, degrees of freedom and *P* value noted<br>*Give P values as exact values whenever suitable.*
☒	☐	For Bayesian analysis, information on the choice of priors and Markov chain Monte Carlo settings
☒	☐	For hierarchical and complex designs, identification of the appropriate level for tests and full reporting of outcomes
☒	☐	Estimates of effect sizes (e.g. Cohen's *d*, Pearson's *r*), indicating how they were calculated

Our web collection on statistics for biologists contains articles on many of the points above.

Software and code

Policy information about availability of computer code

Data collection	Thermo Scientific EPU Software v3.2.0.4776<br>Bruker Topspin v3.0
Data analysis	Prism 7.04 - for data plotting and analysis<br>Phenix v1.20.1-4487 - Structural refinement software suite<br>PyMol v3.0.1 - Molecular graphics software<br>Coot v0.9.8.92 EL- Structural model building<br>cryoSPARC v4.5.3<br>Chimera X 1.8<br>Bruker Topspin v3.0

For manuscripts utilizing custom algorithms or software that are central to the research but not yet described in published literature, software must be made available to editors and reviewers. We strongly encourage code deposition in a community repository (e.g. GitHub). See the Nature Portfolio guidelines for submitting code & software for further information.

Data

Policy information about availability of data

All manuscripts must include a data availability statement. This statement should provide the following information, where applicable:

- Accession codes, unique identifiers, or web links for publicly available datasets
- A description of any restrictions on data availability
- For clinical datasets or third party data, please ensure that the statement adheres to our policy

The coordinates and the maps for SLC35B1 have been deposited in the Protein Data Bank (PDB) and Electron Microscopy Data Bank (EMD) with entries 9GSL / EMD-51551 (WT apo), 9GRZ / EMD-51529 (WT AMP-PNP), 9I20 / EMD-52578 (WT ADP), 9GRY / EMD-51528 (Q113F AMP-PNP), 9GS7 / EMD-51541 (E33A- AMP-PNP), 9GS5 / EMD-51539 (E33A-ADP-OF) and 9GS3 / EMD-51538 (E33A-ADP-IF), respectively.
Correspondence and request for materials should be addressed to D.D. (ddrew@dbb.su.se).

Research involving human participants, their data, or biological material

Policy information about studies with human participants or human data. See also policy information about sex, gender (identity/presentation), and sexual orientation and race, ethnicity and racism.

Reporting on sex and gender	n/a
Reporting on race, ethnicity, or other socially relevant groupings	n/a
Population characteristics	n/a
Recruitment	n/a
Ethics oversight	n/a

Note that full information on the approval of the study protocol must also be provided in the manuscript.

Field-specific reporting

Please select the one below that is the best fit for your research. If you are not sure, read the appropriate sections before making your selection.

☒ Life sciences ☐ Behavioural & social sciences ☐ Ecological, evolutionary & environmental sciences

For a reference copy of the document with all sections, see nature.com/documents/nr-reporting-summary-flat.pdf

Life sciences study design

All studies must disclose on these points even when the disclosure is negative.

Sample size	Biochemical assays and were typically performed at least in triplicate (n =3) to ascertain accurate values for data shown. Statistical methods were not used to determine sample size, but the number of repeats were chosen based on standard practice in the transporter biochemistry community, and were sufficient to calculate standard deviations or standard error.
Data exclusions	No data was excluded.
Replication	All biochemical assays were repeated at least 3 times and the results were reproduced each time.
Randomization	Randomization was performed for calculating the Fourier-shell correlation of half-maps.
Blinding	No blinding was carried out for biochemical and structural analysis as this is not applicable as the analysis does not include subjects.

Reporting for specific materials, systems and methods

We require information from authors about some types of materials, experimental systems and methods used in many studies. Here, indicate whether each material, system or method listed is relevant to your study. If you are not sure if a list item applies to your research, read the appropriate section before selecting a response.

Materials & experimental systems

n/a	Involved in the study
☐	☒ Antibodies
☐	☒ Eukaryotic cell lines
☒	☐ Palaeontology and archaeology
☐	☒ Animals and other organisms
☒	☐ Clinical data
☒	☐ Dual use research of concern
☒	☐ Plants

Methods

n/a	Involved in the study
☒	☐ ChIP-seq
☒	☐ Flow cytometry
☒	☐ MRI-based neuroimaging

Antibodies

Antibodies used	A mouse monoclonal antibody (YN4027) was raised against human SLC35B1.
Validation	The sequence of the Fab YN4027 was determined via standard 5′-RACE using total RNA isolated from hybridoma cells.

Eukaryotic cell lines

Policy information about cell lines and Sex and Gender in Research

Cell line source(s)	NS-1 myeloma was purchased from ATCC (cat# TIB-18). The HCT 116 (CCL-247 ™) cell line was purchased from ATCC. Sf9 was purchased from ThermoFisher Scientific (cat# 11496015).
Authentication	HCT116 cells were authenticated by STR profiling. Sf9 cell lines were authenticated by the manufacturers and no further authentication was performed.
Mycoplasma contamination	HCT116 cells were tested by PCR. Sf9 were not tested.
Commonly misidentified lines (See ICLAC register)	No commonly misidentified cells were used in this study.

Animals and other research organisms

Policy information about studies involving animals; ARRIVE guidelines recommended for reporting animal research, and Sex and Gender in Research

Laboratory animals	Female MRL/lpr mice, 6 weeks of age, were maintained at temperature and humidity ranges of 22 to 26oC and 40% to 60%, respectively under a 14-h light, 10-h dark cycle.
Wild animals	No wild animals were used in this study.
Reporting on sex	Sex-based analyses were not performed.
Field-collected samples	No field collected samples were used in this study.
Ethics oversight	All animal experiments conformed to the guidelines of the Guide for the Care and Use of Laboratory Animals of Japan and were approved by the Kyoto University Animal Experimentation Committee.

Note that full information on the approval of the study protocol must also be provided in the manuscript.

Plants

Seed stocks	*Report on the source of all seed stocks or other plant material used. If applicable, state the seed stock centre and catalogue number. If plant specimens were collected from the field, describe the collection location, date and sampling procedures.*
Novel plant genotypes	*Describe the methods by which all novel plant genotypes were produced. This includes those generated by transgenic approaches, gene editing, chemical/radiation-based mutagenesis and hybridization. For transgenic lines, describe the transformation method, the number of independent lines analyzed and the generation upon which experiments were performed. For gene-edited lines, describe the editor used, the endogenous sequence targeted for editing, the targeting guide RNA sequence (if applicable) and how the editor was applied.*
Authentication	*Describe any authentication procedures for each seed stock used or novel genotype generated. Describe any experiments used to assess the effect of a mutation and, where applicable, how potential secondary effects (e.g. second site T-DNA insertions, mosiacism, off-target gene editing) were examined.*
